# Haptic Connection to a Partner Enhances Motor Learning Retention in Older Adults: Implications for Multiplayer Robotic Rehabilitation

**DOI:** 10.1109/TNSRE.2026.3742242

**Published:** 2026

**Authors:** Erica L. Waters, Nova Y. Meng, Natalia Stroutinsky Sobotka, Frances S. Shofer, Pamela Z. Cacchione, Rochelle J. Mendonca, Michelle J. Johnson

**Affiliations:** Department of Mechanical Engineering and Applied Mechanics, University of Pennsylvania, Philadelphia, PA 19104 USA; Department of Bioengineering, University of Pennsylvania, Philadelphia, PA 19104 USA; Penn Medicine Rehabilitation, Philadelphia, PA 19146 USA; Department of Emergency Medicine, Perelman School of Medicine, University of Pennsylvania, Philadelphia, PA 19104 USA; School of Nursing, University of Pennsylvania, Philadelphia, PA 19104 USA; Department of Rehabilitation and Regenerative Medicine/Programs in Occupational Therapy, Columbia University, New York, NY 10027 USA; Department of Mechanical Engineering and Applied Mechanics, University of Pennsylvania, Philadelphia, PA 19104 USA, also with the Department of Bioengineering, University of Pennsylvania, Philadelphia, PA 19104 USA, and also with the Department of Physical Medicine and Rehabilitation, University of Pennsylvania, Philadelphia, PA 19104 USA

**Keywords:** Rehabilitation robotics, aging, serious games, robot-based haptic dyads

## Abstract

There is a growing interest in understanding how haptic communication between individuals can improve motor learning, particularly in the context of robot-assisted neurorehabilitation. Multi-user robotic systems can haptically connect patients, caregivers, and/or clinicians, providing an implicit communication channel and increasing engagement through multiplayer games. Previous studies of haptic communication and motor learning, however, have focused on pairs of healthy young adults completing relatively simple robot-based tasks. We present a study investigating older adults learning a 1-degree-of-freedom robot-based pursuit tracking task under two conditions: solo and dyad. We evaluated the evolution of motor performance in 38 trials with 1-week follow up. We found no between-group differences in tracking error at any time point. Within-groups, we observed significantly lower tracking error at both the same-day assessment and 1-week retention compared with baseline for both the more skilled and less skilled members of the dyads, whereas the solo group showed no significant improvements across trials. Learning curve analysis further demonstrated superior retention in the less skilled dyad partners relative to the solo group. During the interaction trials, the less skilled partner decreased their tracking error while the more skilled partner maintained performance. We also note exaggerated movements and increased effort from the more skilled partners in the dyads, which may suggest the emergence of a leader-follower dynamic. Together, these findings indicate that training with a haptic connection to a partner yields modest benefits for motor learning in older adults, with the strongest evidence for improved retention among the less skilled partners.

## I. Introduction

HAPTIC feedback is foundational to robot-assisted rehabilitation, but how best to provide that feedback remains an open question. Traditionally, rehabilitation robots used passive haptic guidance, which moves the patient’s limb along a desired path, and later assistance-as-needed control, which only provides as much assistance as required to complete the task [1]. Other approaches to robotic control for rehabilitation have focused on amplifying error and adding haptic disturbance [2], [3]. These approaches can increase motor variability, an essential component in motor learning [4]. While existing control techniques present valid methods of communicating task goals and providing instruction, they lack the depth of information available in haptic communication between humans [5].

A better approach may lie in understanding and replicating how humans interact. In neurorehabilitation, physical touch between therapist and patient can serve as an implicit communication channel for providing instruction, directing attention, and offering comfort [6], [7]. Haptic feedback between individuals can be an effective instructional resource with participants in partnered stepping tasks communicating movement goals and effectively coordinating their actions through haptic interaction alone [8], [9]. Likewise, haptic communication can serve a social role, helping to build relationships and communicate care [10]. Translating the nuance of human–human haptic interaction to a robotic platform requires simplifying the interaction, particularly studying how human partners communicate during motor training and if that communication benefits motor learning [11].

Robot-based haptic dyads—defined as “at least two humans collaborating to complete a task by exchanging forces through interaction with a robotic interface” [12]—represent a paradigm for understanding human haptic communication. Experiencing a haptic connection to a human partner can enhance motor learning compared with training without haptic feedback [13], [14], [15], [16] or training with haptic guidance via connection to the target position [15], [17]. In the context of community-based exercise, robot-based haptic dyads could be a valuable tool for promoting healthy aging. Multi-domain interventions that combine physical activity, cognitive training, and social networking show promise for promoting cognition and functional activity [18], and multiplayer robot-assisted serious games have been proposed for increasing motivation and engagement in rehabilitation while maintaining individualized support [19]. We propose, therefore, that understanding how haptic connection to a partner influences motor learning in older adults could provide insight into multiplayer robotic platforms for community-based exercise and neurorehabilitation.

Current evidence supporting robot-based haptic dyads for motor learning, however, is limited to healthy young adults, as described in Section II. It is unclear if older adults would experience the same benefits from robot-based haptic dyads as young adults. Cognitive abilities such as processing speed, selective attention, and working memory tend to decline with age while other abilities such as visuospatial abilities and vocabulary remain unchanged [20]. Likewise, haptic performance may decline with age and may be related to cognition [21]. In goal-directed motor tasks, older adults exhibit slower movements with higher variability and may rely on slower feedback control processes rather than feedforward control [22], [23]. These differences in motor and cognitive abilities could influence how older adults interact and learn in robot-based haptic dyads. In the present study, we investigate how older adults with no neurological impairments learn and retain a robot-based task both with and without a haptic connection to a partner.

## II. Related Work

The past decade has seen a growing interest in the influence of human-human haptic communication on motor learning. In a recent review paper, we investigated the effects of robot-based haptic dyads on motor learning [12]. In the review, we found that task complexity and relative partner skill level influenced motor learning in a robot-based haptic dyad. Task complexity can vary based on degrees of freedom (DoF), speed, feedback (i.e. visual and haptic), participant skill level, and cognitive load. Simple tasks such as 1-DoF target tracking do not show significant same-day improvements in motor learning among healthy young adults who train in a robot-based haptic dyad compared to individuals who train in a solo condition with no haptic feedback, but working in a dyad does improve motor learning retention [17]. However, complex tasks, such as handwriting [13] or a simulated biopsy task [14] show improved learning with a haptic connection to a partner. Notably, medium complexity tasks (i.e. 2-DoF tracking tasks) have yielded mixed results with some studies showing improved learning for the participants in a robot-based haptic dyad compared to solo [15], [16] and others showing no significant differences [24], [25]. In summary, evidence from healthy young adults suggests that training with a haptic connection to a partner increases retention and may increase motor learning only when the task is sufficiently challenging.

Relative skill level may also influence motor learning. This evidence is most apparent in pairings between individuals with no prior exposure to a given task (“novices”) and individuals who have prior training (“experts”). For example, novice-novice dyads exhibited greater motor learning than novice-expert dyads in both a target tracking task [15] and a nonlinear spring balancing task [26], [27]. Pairing a novice with an expert may induce slacking on the part of the novice or limit task exploration and motor variability, thus reducing motor learning [26]. Conversely, within a novice-novice dyad, a haptic connection to a more skilled novice may benefit motor learning. Research shows that the less skilled partner learns more than the more skilled partner in a tracking task [24], [28]. Furthermore, training with a haptic connection to a partner increases motor learning compared to training with haptic guidance [15], [17]. This increase in learning when training with a reactive partner compared to haptic guidance may be due to the increased variability of a human partner [17], [29]. Together, these results suggest that interacting with a partner during training goes beyond motion assistance; rather, haptic communication occurs between partners and may promote greater motor variability given that the partners in the dyad are appropriately matched in terms of experience.

Although many robot-based haptic dyad studies cite the potential to apply their results to neurorehabilitation, most studies are limited to healthy young adults, which may lead to performance ceiling effects [12], [30]. Current literature on robot-based haptic dyads in individuals with neurological impairments is limited to chronic stroke: two pilot studies demonstrating feasibility of therapist-patient exoskeleton interaction [31], [32] and two pilot studies investigating same day improvements [28], [29]. Despite preliminary evidence supporting the feasibility of robot-based haptic dyads for neurorehabilitation, relatively little has been done to understand how older adults, who have the highest need for neurorehabilitation, respond to haptic communication with a partner. To our knowledge, the only study investigating motor learning in a robot-based haptic dyad that specifically included older adults, age 50 years or more, is our previously published pilot study, which recruited six healthy older adults and three older adults with post-stroke motor and cognitive impairments [28]. If robot-based haptic dyads are to be clinically applicable for neurorehabilitation, we must first establish its efficacy for neurological populations and older adults.

## III. Methods

### A. Participants and Conditions

Adults age 50 and older with no reported neurological disorders were recruited to complete the 3-day study. Participants were excluded if they had greater than mild depression—assessed by the Becks Depression Inventory-Fast Screen (score ≥ 4) [33]. This protocol was approved by the Institutional Review Board of the University of Pennsylvania (Protocol no. 851418).

### B. Experimental Protocol

As shown in Figure 1D, participants completed three sessions: clinical assessment, robotic training, and retention assessment. In session one, participants completed clinical assessments with a trained occupational therapist. In session two, participants completed the baseline block, which consists of four solo trials with the robot, followed by usability surveys. They then completed a training block of 30 dyad (or solo) trials depending on their training condition. After training, they completed the usability surveys again. To conclude the robot training day, they completed an assessment block of 4 solo trials. Six to fourteen days after the training session, participants completed the retention block, which consisted of 8 solo trials with the robot.

#### 1) Clinical Assessments:

Although participants reported no neurological disorders, a licensed occupational therapist administered a battery of motor and cognitive assessments. These assessments were essential because many older adults have undiagnosed motor and cognitive impairments [34], [35]. The motor assessments included the Box and Block Test (BBT) and the Grooved Pegboard (GP). BBT is a test of gross manual dexterity that tasks participants to move cubes across a partition—the score is determined by the number of blocks transferred in one minute with more blocks indicating higher gross manual dexterity [36]. GP is a manipulative dexterity test that measures the length of time required for a participant to place 25 grooved pegs into keyed holes [37]. The cognitive assessments included Color Trails 1 and 2 (CT1, CT2) and the Montreal Cognitive Assessment (MoCA). The Color Trails are trail making tests of cognitive function where performance is assessed as time to complete the task. CT1 tests visual attention and sequencing, and CT2 tests visual-spatial skills, selective attention, and mental flexibility [38]. MoCA is a cognitive screening used to detect mild cognitive impairment (MCI)—a score of 26 or greater is considered cognitively intact while a lower score indicates MCI [39].

#### 2) Robotic System:

For motor training, participants used the TheraDyad robotic system (Figure 1A), which consists of two TheraDrive robots. The TheraDrive robot is a 1-DoF system that was developed as an affordable system for robot-assisted arm therapy [40]. The current version of the TheraDrive (now called THEABot, Recupero Robotics LLC) is a reconfigurable system, which can provide comprehensive arm exercise through interchangeable handles and intentional positioning [41]. For the purposes of this study, we positioned the robot vertically with the workspace along the user’s transverse plane (Figure 1B). This position involves horizontal abduction/adduction of the shoulder and mimics exercises with an arm skate, which are common in neurorehabilitation [41]. Each robot has a dedicated monitor to display the task. A privacy screen between the two robots limits visual information about the partner.

TheraDyad connects the two THEABot robots using a position-position controller that renders a virtual spring-damper link between the two robots. For stiffness, we selected a linear equivalent stiffness of 150N/m or 8.284 Nm/rad. Greater detail concerning this controller is provided in our prior publication [28]. To better mimic a group therapy session with TheraDyad, we elected to provide constant coupling between robots for the training trials in the dyad condition. No additional perturbation forces were applied in order to keep communication across the haptic link as transparent as possible.

#### 3) Robot-Based Task:

Participants learned a standardized 1-DoF pursuit tracking task that maps the rotational motion of the robot in the horizontal plane to vertical, linear movement on the screen, as shown in Figure 1B–C. The task was considerably faster than comparable tasks reported in the literature [15], [17], [24] with a mean velocity of 104 *deg*/*s* or 0.43 *m*/*s*. Participants trained with their non-dominant hand and were not told the mapping before training. They controlled a square shaped cursor and were instructed to keep the cursor inside of a gray rectangular target. For additional feedback, the cursor changed color from yellow to magenta when inside of the target. Each trial lasted fifteen seconds. In the dyadic conditions, the cursor position was placed at the midpoint between the two robot positions. Participants were told that they may experience forces during the training but were naive to the source of these forces, i.e. haptic connection to a partner. Participants could not see their partner during training and were instructed not to speak to one another during the experiment.

#### 4) User Experience Surveys and Interview:

As shown in Figure 1D, participants completed usability surveys and a short interview. More information about the surveys is provided in our previously published pilot study [28]. Analysis of survey and interview responses is beyond the scope of this report but was presented in our recent conference abstracts [42], [43].

### C. Data Analysis

Dyad participants were separated into two groups for analysis: more skilled and less skilled. For each dyad, the partner with lower tracking error in the baseline block was labeled “more skilled” while the partner with higher tracking error was labeled “less skilled.” These labels were determined after the experiment, so the participants and researchers were naive to the labels during training.

#### 1) Outcome Measures:

We defined three outcome metrics to assess task performance in terms of accuracy, efficiency, and effort.

##### a) Root mean square error (RMSE):

Accuracy was defined as RMSE across each trial with given number of samples (n), calculated according to Equation 1.

(1) $$RMSE=\sqrt{\frac{1}{n}\sum_{i=0}^{n}{\left(\theta_{t,i}-\theta_{h,i}\right)}^{2}}$$

Here, $\theta_{t}$ represents the desired or target position and $\theta_{h}$ represents the position of the robot controlled by the human operator; both values are represented in degrees. A higher value of RMSE indicates worse performance, i.e., greater tracking error.

##### b) Normalized distance (ND):

Movement efficiency was assessed with ND, which is the total distance traveled divided by the distance of the desired trajectory and is calculated according to Equation 2.

(2) $$ND=\frac{\sqrt{\sum_{i=1}^{n}{\left(\theta_{h,k}\;-\;\theta_{h,i-1}\right)}^{2}}}{\sqrt{\sum_{i=1}^{n}{\left(\theta_{t,i}\;-\;\theta_{t,i-1}\right)}^{2}}}$$

A ND value of one indicates a perfectly efficient movement. A value greater than one indicates that the participant moved more than the task requires, which may indicate inefficient movements. A value below one indicates that the participant moved less than the task requires, which may indicate a failure to accomplish the task.

##### c) Mean absolute power (MAP):

Effort was quantified as absolute power (MAP) for each trial, which is calculated according to Equation 3.

(3) $$MAP=\frac{1}{n}\sum_{i=1}^{n}|\tau_{i}\cdot {\dot{\theta }}_{i}|$$

Here, τ denotes torque and $\dot{\theta }$ denotes angular velocity. Higher values of MAP indicate greater effort, while lower values indicate less effort.

### 2) Statistical Analysis:

The outcome measures (RMSE, ND, MAP) were calculated for all trials. To examine changes over time, we split the data into six distinct time points: baseline (mean of trials 1–4), training 1 (mean of trials 5–14), training 2 (mean of trials 15–24), training 3 (mean of trials 25–34), assessment (mean of trials 35–38), and retention (mean of trials 39–46). We performed three 2-way analysis of variance (ANOVA) tests in repeated measures where the grouping factor was group (n= 3, more skilled, less skilled, solo) and the repeated measure was trial (n= 6, baseline, training 1, training 2, training 3, assessment, retention) on RMSE, ND, and MAP. Data were confirmed to be normally distributed before performing an ANOVA using the Shapiro-Wilk test. While RMSE and ND showed no significant deviations from normality, MAP displayed four statistically significant (*p* < 0.05) deviations from normality (training 1, training 2, and training 3 in Dyad (LS); training 3 in Dyad (MS)). Visual inspection of Q-Q plots indicated that these deviations were driven by single outliers rather than systematic departures from normality. Given the robustness of repeated-measures ANOVA to minor violations of normality, we retained all data and proceeded with the analysis. To adjust for multiple comparisons within each outcome measure, post-hoc Tukey Honestly Significant Difference (HSD) pairwise tests were performed. No adjustments were made across outcome measures for this exploratory study.

### 3) Learning Curve Analysis:

To identify learning behavior across groups and within individual participants, we utilized learning curves. When an individual is first exposed to a new task, they experience rapid improvement that decreases over time; this learning curve approximates an exponential function [44]. When an individual is brought back after a period of approximately one-week, their learning approximates the same shape with fewer errors in the first trial and a more rapid relearning rate. Given the interaction component for the dyad participants, we modified the standard exponential learning curve equation to include a constant training offset $\left(\beta_{t}\right)$ that was only applied during the training trials. This addition to the model helped to account for any instantaneous improvement that participants experience during the training trials as a result of interacting with a partner. The exponential decay functions with training offset are represented by the piecewise function in Equation 4.

(4) $$y=\left\{\begin{array}{ll} \alpha -c\cdot e^{-\ell \cdot (t-1)} & t<5,\;35\leq t\leq 38\\ \alpha -c\cdot e^{-\ell \cdot (t-1)}+\beta_{t} & 5\leq t\leq 34\\ \alpha -c_{r}\cdot e^{-\ell_{r}\cdot (t-39)} & t>38 \end{array}\right.$$

In Equation 4, the asymptote, or final performance, is represented by the variable α where a lower value represents better final performance. The total improvement, or difference between the first and last trial (t) on day one (t≤38), is represented by the variable c; a larger value indicates more learning occurred. The learning rate, or rate of improvement across trials on day one (t≤38), is represented by the variable ℓ where a larger value indicates faster learning. The training offset variable $\left(\beta_{t}\right)$ is a constant that is only applied during the training trials (5≤t≤34), and a negative value indicates temporary improvement during the training trials. The total forgetting after the one-week retention is represented by the variable $c_{r}$, and the relearning rate is represented by the variable $\ell_{r}$. A smaller total forgetting ($c_{r}$) indicates better retention, and a larger relearning rate ($\ell_{r}$) indicates faster relearning.

To represent learning curves, we fit the learning curve in Equation 4 to tracking error (RMSE) across trials using a nonlinear mixed effects model in the R Linear and Nonlinear Mixed Effects Models (nlme) package [45]. Variables from Equation 4 are fit as group-level fixed-effects $\left(\ell ,\ell_{r}\right)$ and effects $\left(\alpha ,\beta_{t},c,c_{r}\right)$. This structure allows observation of the overall pattern of behavior within the three groups while accounting for individual differences. We report the estimated value and 95% confidence interval for all fixed-effects by group; significance is assessed using Wald t-tests provided by the nlme summary output.

Kinematic analysis was completed in Python. All statistical analyses were performed using R Statistical Software (v4.4.1; R Core Team 2024) [46].

## IV. Results

The final study included 21 healthy older adult participants (age range of 51–80 yrs, average age 65.3 ± 7.1 yrs; 11F/10M), solo (n = 7) and dyad (n = 14). There were no differences in demographics or metrics when comparing the 3 groups (Table I). As shown in Figure 2, the participant with lower tracking error at baseline was designated the more skilled (MS) partner while the partner with greater error was designated the less skilled (LS) partner. Skill differences within the dyads varied: the percent difference in baseline RMSE between dyad partners had a mean of 15.2% ± 10.4% and ranged from 0.9% to 29.0%. Table II shows the group results for all performance metrics across trials.

### A. Root Mean Square Error (RMSE)

There were no differences in RMSE at each time point between groups (Figure 3A). Within groups, however, there were differences in RMSE across trials (Figure 3D). As shown in Table II, the less skilled dyad group showed higher RMSE at baseline compared to the three training trials, assessment, and retention. The more skilled dyad group similarly showed higher RMSE at baseline compared to assessment and retention. The solo group showed no differences across trials.

### B. Normalized Distance (ND)

As shown in Figure 3B, the more skilled dyad group demonstrated higher ND compared to the solo group during training 1 (Δ = −0.566; 95%*CI* : −1.00, −0.128; *p* = 0.011), training 2 (Δ = −0.554; 95%*CI* : −1.01, −0.0952; *p* = 0.017), and training 3 (Δ = −0.600; 95%*CI* : −1.02, −0.177; *p* = 0.005). Likewise, individual groups demonstrated differences in ND across trials (Figure 3E). Compared to baseline, the more skilled dyad group showed higher ND in the three training trials; this increase was partially maintained at assessment and retention although the values were not significantly different from baseline. Likewise, the less skilled dyad group showed lower ND at baseline compared to the training trials, but this difference only reached significance for the third training block. The solo group showed no change in ND across trials. Table II shows the numerical results for these comparisons.

### C. Mean Absolute Power (MAP)

Compared to solo, both the more skilled and less skilled dyad groups displayed higher MAP during the training trials (Figure 3C). The more skilled group showed the largest difference in MAP compared to solo at training 1 (Δ = −4.16; 95%*CI* : −6.85, −1.46; *p* = 0.003), training 2 (Δ = −4.47; 95%*CI* : −7.24, −1.71; *p* = 0.002), and training 3 (Δ = −4.00; 95%*CI* : −6.39, −1.62; *p* = 0.001), while the less skilled group showed meaningful but slightly lower differences with solo at training 1 (Δ = −2.76; 95%*CI* : −5.46, −0.067; *p* = 0.044), training 2 (Δ = −2.97; 95%*CI* : −5.73, −0.202; *p* = 0.034), and training 3 (Δ = −2.81; 95%*CI* : −5.19, −0.425; *p* = 0.020). Within groups, there were differences in the more skilled and less skilled dyad groups across trials (Figure 3F). Compared to baseline, both dyad groups displayed higher MAP in all three training trials. The solo group showed no change in MAP across trials. Table II shows the numerical results for these comparisons.

### D. Learning Curves

Figure 4 shows the learning curves fitted to each group, and Table III presents the fixed-effects estimates for the parameters that characterize these curves as defined in Equation 4. Asymptote, total change, and learning rate showed no differences between groups. Training offset, however, was lower for the less skilled group compared to solo. The less skilled group also demonstrated less forgetting and lower relearning rate compared to solo, while the more skilled group demonstrated similar forgetting and relearning rate as solo.

## V. Discussion

We investigated how older adults learn a 1-DoF robot-based pursuit tracking task both with and without a haptic connection to a partner. While other studies have investigated learning in robot-based haptic dyad, this is the first study to focus exclusively on older adults who may have motor and cognitive deficits. The presence of impairments, e.g. MoCA < 26, in the included population results in a larger distribution of task performance levels than previously observed in other studies. Furthermore, many neurological conditions, such as Parkinson’s disease and stroke, are more likely to affect older adults. It is therefore essential to understand how aging may influence motor performance and learning in a robot-based haptic dyad intended for upper-limb rehabilitation.

### A. Dyad Partners Improve or Maintain Performance During Interaction

Consistent with the literature [15], [24], [47], [48], our results indicate that a connection to a partner allows the less skilled partner to improve their performance and the more skilled partner to maintain their performance during interaction trials. The less skilled group is the only group to show a substantial decrease in tracking error, as measured by RMSE, in the training blocks compared to baseline (Figure 3D). Likewise, the training offset parameter of the learning curve mixed-effects model shows a difference between the less skilled dyad group compared with the solo group (Table III). The more skilled dyad group does not show a difference in RMSE between baseline and training (Figure 3D). Together, these results indicate that working in a dyad provides an immediate boost in performance for the less skilled partner and allows the more skilled partner to maintain their performance.

### B. The More Skilled Partner Leads During Interaction

As expected, both dyad partners demonstrate substantially greater effort, as measured by MAP, during training compared with the solo group (Figure 3C), and this MAP at training is greater than their baseline (Figure 3F). This increased effort is an expected result under the interaction condition due to interaction forces, but this increase in forces may also represent the emergence of a haptic communication channel [49]. The more skilled dyad partner, however, appears to increase their MAP more than their less skilled partner with a training MAP that is much higher than their MAP at assessment and retention. This dominance of the more skilled partner is also observed in ND. During the training blocks, the more skilled dyad group increased their distance traveled (Figure 3E) while maintaining tracking error (Figure 3D), traveling more to achieve the same error. Figure 2 shows how the more skilled partner may exaggerate movements during training by increasing the distance that they move compared with their baseline performance. The less skilled dyad partner also showed greater ND in the training session compared to their baseline (Figure 3E), but only the more skilled partner moved more than the task requires (ND > 1) and moved significantly more than the solo group (Figure 3B). Together, this heightened MAP and ND indicate that the more skilled partner dominates the interaction during the dyad trials.

We note, however, that dyads had different skill disparities between partners. While most dyads exhibited large differences in baseline RMSE, two of the dyads had partners with functionally equivalent baseline performance, i.e. percent difference in baseline RMSE below 10%. In these dyads, the designated more skilled partner was not necessarily more dominant. In fact for the dyad with the smallest difference between partners (0.9% difference in baseline RMSE), the partner designated as “less skilled” dominated the interaction with the less skilled partner demonstrating higher MAP and ND (Training 1: MAP=9.87, ND=1.42; Training 2: MAP=9.54, ND=1.47; Training 3: MAP=9.57, ND=1.41) relative to the more skilled partner (Training 1: MAP=6.31, ND=0.96; Training 2: MAP=6.51, ND=0.91; Training 3: MAP=6.50, ND=0.94). Nevertheless, dyads with similar skill levels still demonstrated a clear difference in dominance. For dyads with similar baseline tracking error, it is difficult to predict which partner will dominate the interaction. The observed dominance difference in dyads with similar baseline skill levels could be related to other factors such as motor variability [17], which is related to capacity for motor learning [4], [50], or an innate dominance tendency [51], which could be related to intrinsic motivation as observed in preference for competitive games [52].

Dominance of one partner over another in dyadic interactions is commonly observed in the literature [48], [51], [53], [54]. One explanation for the dominance of the more skilled partner is compensation for the less skilled partner’s poor performance. In this view, the partners may ignore the haptic information and choose to pursue the target according to their own strategy. While the more skilled partner exaggerates their movement in order to reach the target, thus leading to greater ND and MAP as they fight against their partner, the less skilled partner is not strong enough to fully resist the more skilled partner’s movement, leading to temporarily decreased RMSE and increased ND. The explanation that the partners ignored the interaction, however, is inconsistent with the results and does not explain why the less skilled partner was able to maintain their improved performance after the interaction is removed (Figure 3D). Likewise, computational models of healthy dyadic interactions indicate that partners do not ignore the haptic signal from a partner [47]. While the more skilled partner may compensate for their partner during the interaction, it is unlikely that they ignore the haptic signal.

Another interpretation is that the more skilled partner assumes a “leader” role while the less skilled partner assumes a “follower” role. Leader-follower roles have been observed in robot-based haptic dyads before due to asymmetrical task dynamics [55] or asymmetrical information [56]. In the present study, the task is symmetrical, but performance levels may be asymmetrical, leading to the natural development of leader and follower roles. This finding aligns with Liu et al. who observed that the more skilled partner in a dyadic symmetrical ball-beam task tended to lead the interaction [54]. Importantly, the less skilled partner is not passive; rather, they follow the more skilled partner to an extent while maintaining their own strategy (Figure 2). The observed leader-follower relationship, therefore, is not a simple follow-the-leader interaction but more akin to a teacher-student interaction. In their role as “teacher,” the more skilled partner may exaggerate their movements in order to better communicate task objectives to the less skilled partner. This interpretation aligns with computational models of dyad interactions, which support that partners communicate goals via the shared haptic channel [47], [48].

This tendency of the more skilled partner to dominate interactions could have implications for robot-based haptic dyads in rehabilitation. When pairing differently skilled partners, such as an older adult paired with a clinician, it may be necessary to balance partner performance through game mechanics [52], [57] or the robotic controller [19]. Preliminary studies in healthy young adults have shown promise for balancing partner effort in differently skilled dyads through an asymmetric connection [58] and supporting motor learning by augmenting haptic connection based on player effort [59]. Furthermore, increasing evidence highlights the need for greater social interaction and activities among both older adults and individuals with stroke [60], [61]. Robot-based haptic dyad interventions can not only promote physical function but also address the social aspect. Robot-based multiplayer games can leverage social interaction in order to increase motivation and exercise intensity [19], [25], but the effectiveness of these interventions depends on factors such as game mode (competitive vs. cooperative) [52], [62], relative partner skill level [57], and familiarity with partner [62], [63]. More work is necessary to understand how to best balance dyad ability level in order to best support motor learning, motivation, and social interaction in rehabilitation.

### C. Training in a Dyad Boosts Motor Learning Retention

Both the less skilled and more skilled dyad groups show lower RMSE at assessment and retention compared with baseline while the solo group shows no significant difference between baseline and assessment or baseline and retention (Figure 3A). Older adults who train in the dyad, therefore, not only demonstrate learning, but also the ability to retain that learning. In contrast, older adults who train in the solo condition demonstrate only modest improvement. Despite these differences, the learning curve analysis did not show significant differences in learning parameters (overall change, learning rate) between either dyad group and the solo group. The less skilled dyad group, however, showed less forgetting compared with the solo group, achieving a near zero forgetting (Table III). The less skilled dyad group was also the only group to show improvement beyond asymptotic performance during the retention day (Figure 4). Likewise, we did not observe significant differences between groups at assessment or retention. Although not significant, the solo group demonstrated worse scores on Box and Blocks relative to both dyad groups and on the Color Trails assessments relative to the more skilled dyad (Table I). The solo group was also somewhat older. Together, these observations may have contributed to the observed modest learning results in the solo group. Finally, we note that there was some variability in timing for the retention day. Due to scheduling conflicts, four participants were not able to return exactly one-week later: two less skilled dyad participants returned 14 days later, one solo participant returned 8 days later, and one more skilled dyad participant returned 6 days later. Nevertheless, our results suggest that training in a robot-based haptic dyad may provide a slight boost to motor learning, particularly in retention for the less skilled partner.

These results are consistent with the literature on healthy young adults that show increased motor learning in a robot-based haptic dyad [13], [14], [15], [16] or increased retention of the motor task [17]. However, our results do not demonstrate increased same day learning for the less skilled partner relative to the more skilled partner, as shown in some of the literature [24], [28]. We also note that not all experiments with healthy participants in haptic dyads show significant same-day improvements in motor learning [17], [24], [25]. Beyond the older age of our participants, one explanation for the observed motor learning difference in our study is the difficulty of the task. Faster movements in tracking tasks are associated with higher error—i.e. higher task difficulty—than slower movements, especially for older adults [64]. Comparing our task to similar tracking tasks reported in the literature, our task (mean velocity of 104 *deg*/*s* or 0.43 *m*/*s*) is considerably faster than studies that report no learning difference between dyad participants and solo participants. Ivanova et al. report a task with a mean velocity of 19 *deg*/*s* (calculated from the provided sinusoidal equation) [17], and Beckers et al. report a mean task velocity of 0.079 *m*/*s* [24]. Ganesh et al., who did observe a significant difference between dyad training and solo training on motor learning, report a mean task velocity of of 0.18 *m*/*s* [15], which is faster than Beckers et al. and slower than our task. Task difficulty, of course, is not just related to velocity; handedness may also affect the difficulty of the task. Participants in this study trained with their non-dominant hand, which may increase task difficulty. In fact, recent understandings of limb dominance suggest that the dominant limb is faster and specialized for specifying direction and trajectory, primarily relying on feedforward control mechanisms, while the non-dominant hand is robust to disturbance and specialized for final positioning, primarily relying on feedback control mechanisms [65], [66]. Using the non-dominant hand rather than the dominant hand to complete a fast-pace, pursuit tracking task most likely increased the task difficulty. The combination of an older population and greater task difficulty creates a larger distribution of participant performance levels, eliminating the potential ceiling effect observed in other studies. This increase in relative task difficulty may explain the observed learning difference for this 1-DoF task compared with similar tasks from the literature.

## VI. Conclusion

We demonstrate that training with a haptic connection to a partner may modestly benefit motor learning in older adults. Although the dyad groups did not achieve significantly better performance relative to the solo group, both the more and less skilled partners in the dyad were able to significantly decrease their tracking error at assessment and retain this improvement at the 1-week post-training. This boost in motor learning retention was particularly apparent in the less skilled group, who showed less forgetting relative to the solo group. We also provide evidence that the more skilled partner may take on a leadership or “teaching” role during interaction. It remains unclear, however, how dominance difference in the dyad scales with the degree of skill disparity between partners. Future work should involve dyads with differing levels of skill disparity and investigate if other factors such as motor variability or innate dominance tendencies influence leader-follower roles during interaction. Future work should also investigate populations with disabilities to better establish the robot-based haptic dyad paradigm for rehabilitation. Likewise, interaction parameters such as stiffness of connection, relative skill between partners, and knowledge of the interaction should be further investigated to better understand the optimal type of interaction for rehabilitation. Finally, future work should investigate the role of socialization in performance and learning in order to best realize robot-based haptic dyads in rehabilitation contexts.

## Figures and Tables

**Fig. 1.**
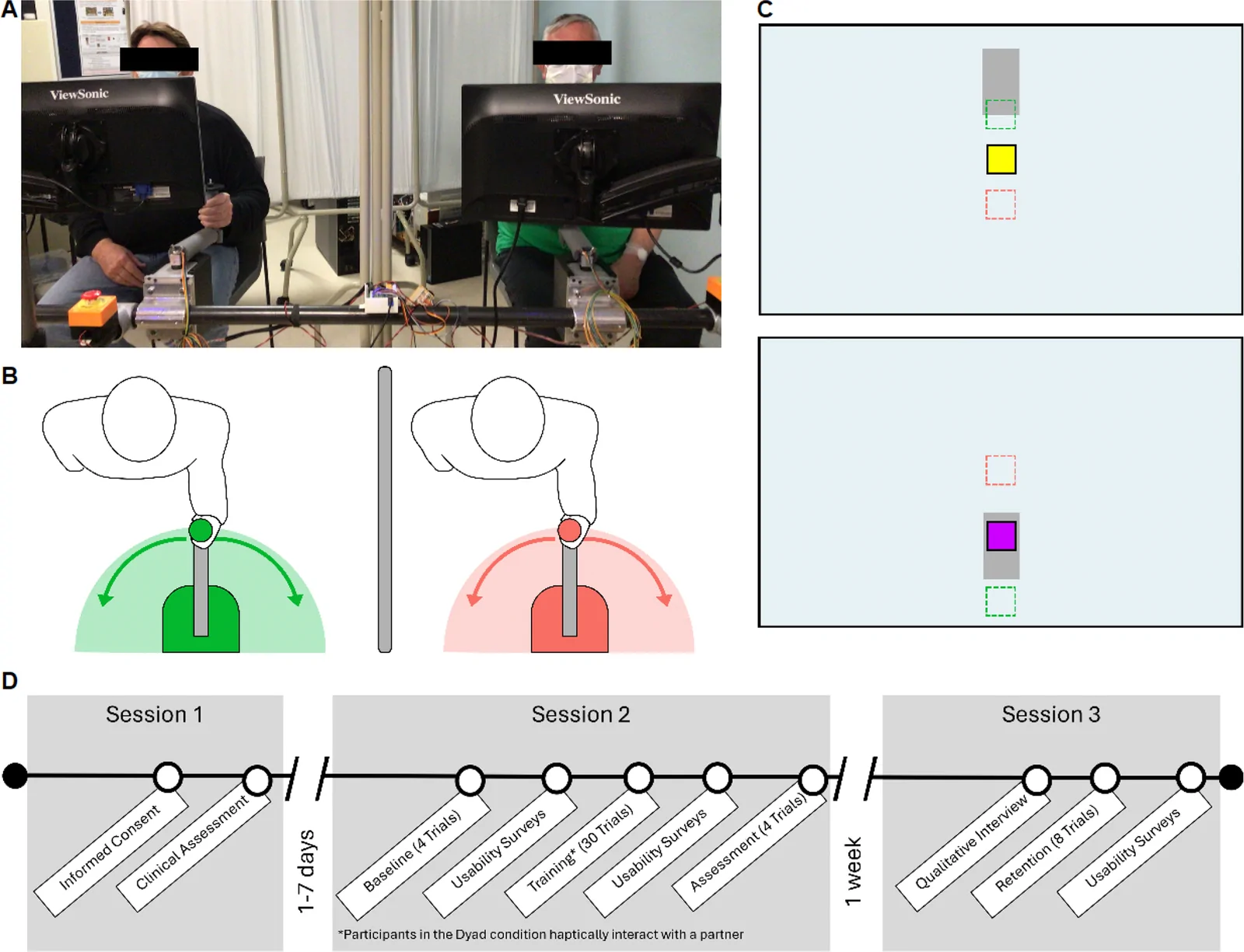
Summarized methods. **A:** Two participants using TheraDyad in the dyadic condition. A privacy screen prevents players from seeing their partner. **B:** Top-down view of experimental setup. Each robot has a half-circle workspace in the horizontal plane. **C:** Motor training task showing the gray target box and the cursor, which is yellow when outside of the box and magenta when inside of the box. The green and red dotted outlined squares (not visible during the trials) represent the cursor position as the average position of the two players’ positions in the dyadic condition. **D:** Timeline of the experimental protocol featuring three session: clinical assessment (session 1), motor training (session 2), and interview/retention assessment (session 3).

**Fig. 2.**
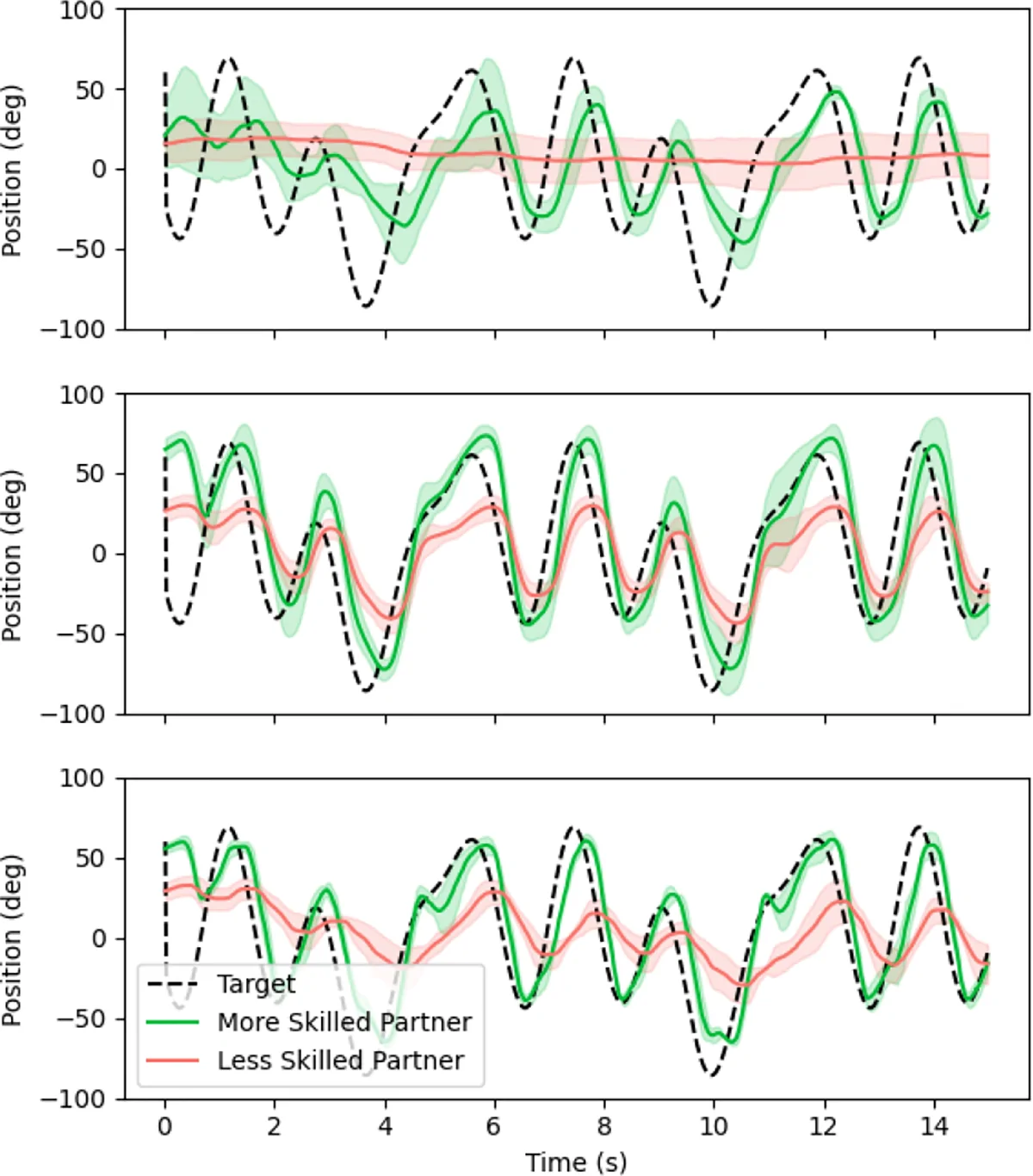
Example mean trajectories for a dyad at baseline (top), training (middle), and assessment (bottom). The target trajectory is denoted as a dotted black line; the standard deviation is denoted as a shaded region.

**Fig. 3.**
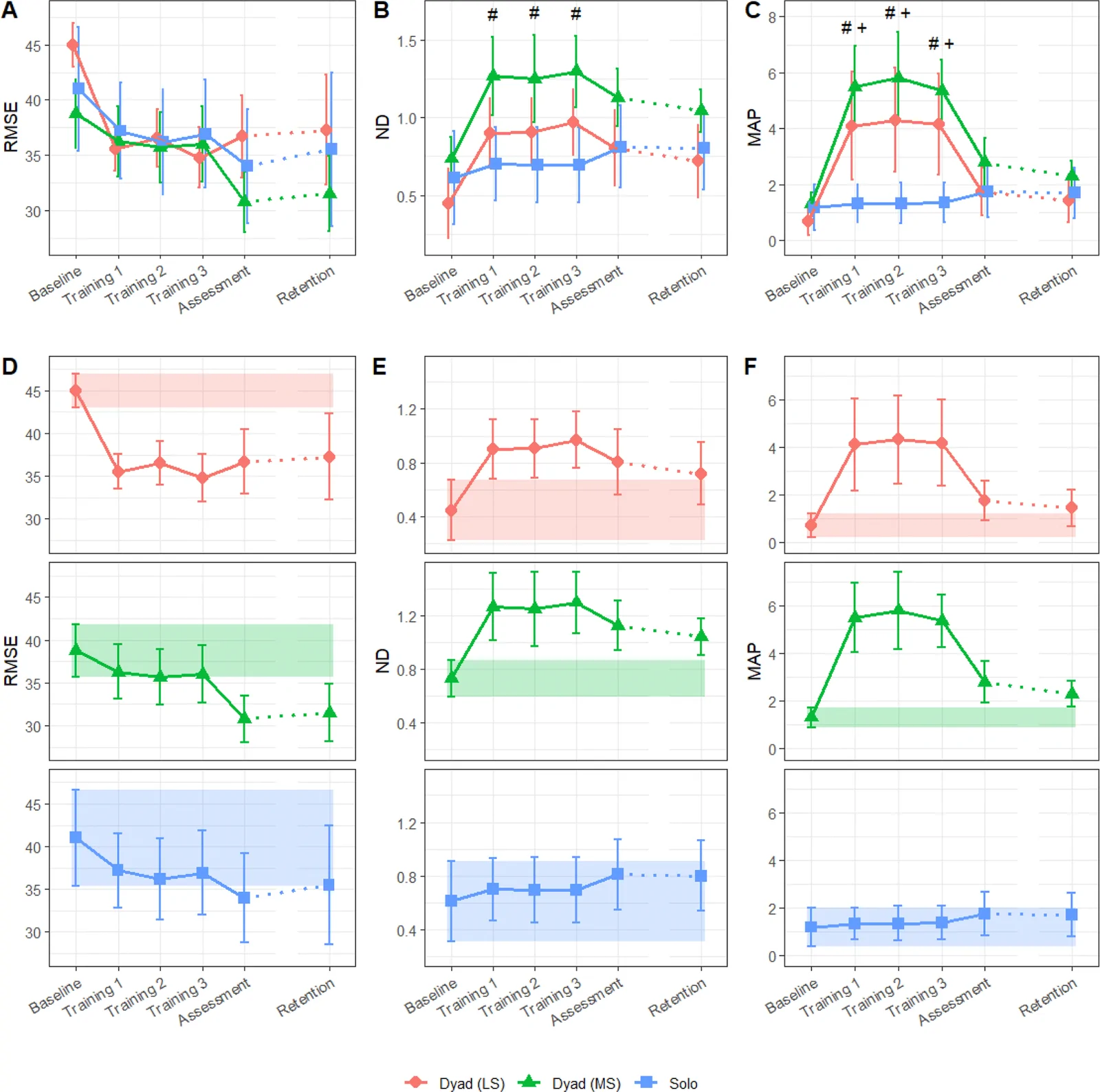
Mean metrics with 95% confidence intervals across six time points. Significant differences (*p* < 0.05) marked as follows: between Dyad Less Skill and Dyad More Skilled (*), between Dyad Less Skilled and Solo (+), and between Dyad More Skilled and Solo (#). Shaded areas represent the 95% confidence interval at baseline. **A:** RMSE comparison between groups. **B:** ND comparison between groups. **C:** MAP comparison between groups. **D:** RMSE differences across trials compared with baseline. **E:** ND differences across trials compared with baseline. **F:** MAP differences across trials compared with baseline.

**Fig. 4.**
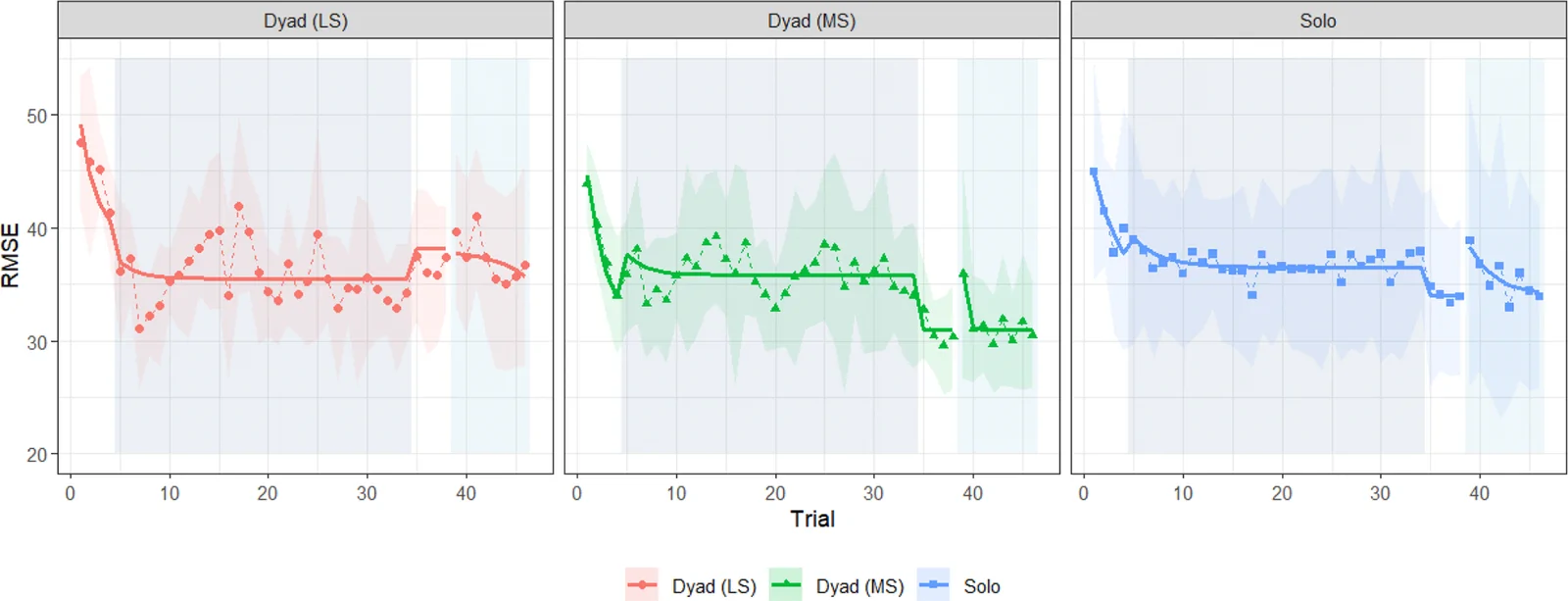
Learning curve results for all participants across three groups: less skilled partner in dyad (LS), more skilled partner in dyad (MS), and solo. Mean RMSE at each trial are represented by points with a dashed line connecting the points. The shaded area around this dashed line denotes the standard deviation of RMSE values at each trial. The solid lines define group-level learning curves, which were fit according to Equation 4. The baseline and assessment blocks include the first and last four trials, respectively. The three training blocks, each consisting of 10 trials, comprise the middle thirty trials and are denoted with a shaded gray box. This shaded area represents the presence of haptic interaction for the dyad participants. The last 8 trials comprise the retention assessment and are denoted with a shaded light blue box.

**TABLE I:** Demographics and Clinical Assessment Scores by Group

Demographic Info or Clinical Score	Group Value (Mean ± Standard Deviation, [Min, Max])		
	Dyad-Less Skilled	Dyad-More Skilled	Solo Group

Age (Years)	63.9 ± 8.2, [54, 78]	63.7 ± 7.9, [51, 72]	68 ± 5.6, [63, 80]
Sex (Female)	5 (71%)	3 (43%)	3 (43%)
Dominant Hand (Right)	5 (71%)	7 (100%)	7 (100%)
Box and Blocks–Dominant (# of blocks)	66.3 ± 10.5, [53, 87]	66.3 ± 8.2, [54, 75]	59.1 ± 8.0, [47, 70]
Box and Blocks–Non-Dominant (# of blocks)	64.3 ± 9.0, [57, 84]	64.9 ± 9.8, [50, 80]	59.0 ± 9.9, [48, 76]
Grooved Pegboard–Dominant (seconds)	74.9 ± 21.6, [46, 114]	66.2 ± 11.7 [53, 88]	70.9 ± 9.4 [59, 82]
Grooved Pegboard–Non-Dominant (seconds)	80.7 ± 27.0, [53, 138]	68.0 ± 5.4, [63, 78]	80.4 ± 16.4, [55, 102]
Color Trails 1 (seconds)	35.4 ± 13.2, [15, 54]	26.4 ± 6.78, [17, 34]	36.9 ± 10.9, [25, 55]
Color Trails 2 (seconds)	94.8 ± 29.9, [42, 127]	68.0 ± 17.5, [50, 92]	96.4 ± 40.7, [47, 177]
Montreal Cognitive Assessment (max 30)	26.1 ± 1.68, [24, 29]	26.7 ± 2.63, [23, 29]	25.9 ± 2.27, [22, 29]

**TABLE II:** Performance Metrics Across Trials and Groups With Change From Baseline

Metric	Trial	Dyad-Less Skilled			Dyad-More Skilled			Solo Group		
		Mean [95% CI]	Δ	p-value	Mean [95% CI]	Δ	p-value	Mean [95% CI]	Δ	p-value

*RMSE*										
	*Baseline*	*45.0 [43.0, 46.9]*			*38.8 [35.7, 41.8]*			*41.0 [35.4, 46.6]*		
	Training 1	**35.5 [33.5, 37.5]**	**−9.41**	**0.003**	36.3 [33.1, 39.4]	−2.50	0.887	37.2 [32.9, 41.6]	−3.81	0.923
	Training 2	**36.5 [34.0, 39.1]**	**−8.42**	**0.011**	35.7 [32.5, 38.9]	−3.07	0.768	36.2 [31.4, 41.0]	−4.81	0.818
	Training 3	**34.8 [32.0, 37.6]**	**−10.2**	**0.001**	36.0 [32.6, 39.4]	−2.75	0.839	36.9 [32.0, 41.8]	−4.10	0.897
	Assessment	**36.7 [32.9, 40.4]**	**−8.28**	**0.013**	**30.7 [28.0, 33.5]**	**−8.00**	**0.016**	34.0 [28.8, 39.2]	−7.00	0.482
	Retention	**37.3 [32.3, 42.3]**	**−7.68**	**0.025**	**31.5 [28.1, 34.9]**	**−7.24**	**0.037**	35.5 [28.6, 42.5]	−5.49	0.723
*ND*										
	*Baseline*	*0.45 [0.22, 0.67]*			*0.73 [0.60, 0.87]*			*0.61 [0.32, 0.91]*		
	Training 1	0.90 [0.68, 1.12]	0.45	0.087	**1.27 [1.02, 1.52]**	**0.53**	**0.016**	0.70 [0.47, 0.93]	0.09	0.997
	Training 2	0.91 [0.69, 1.12]	0.46	0.079	**1.25 [0.97, 1.53]**	**0.52**	**0.021**	0.69 [0.45, 0.94]	0.08	0.998
	Training 3	**0.97 [0.76, 1.18]**	**0.52**	**0.032**	**1.30 [1.07, 1.53]**	**0.56**	**0.009**	0.70 [0.45, 0.94]	0.08	0.998
	Assessment	0.80 [0.56, 1.05]	0.36	0.271	1.13 [0.94, 1.31]	0.39	0.132	0.81 [0.55, 1.07]	0.20	0.897
	Retention	0.72 [0.49, 0.95]	0.27	0.560	1.04 [0.91, 1.18]	0.31	0.354	0.80 [0.54, 1.06]	0.19	0.915
*MAP*										
	*Baseline*	*0.69 [0.18, 1.20]*			*1.31 [0.91, 1.72]*			*1.18 [0.36, 2.00]*		
	Training 1	**4.10 [2.17, 6.03]**	**3.41**	**0.024**	**5.49 [4.03, 6.96]**	**4.18**	**<0.001**	1.34 [0.66, 2.01]	0.15	>0.999
	Training 2	**4.30 [2.44, 6.16]**	**3.61**	**0.015**	**5.81 [4.18, 7.44]**	**4.50**	**<0.001**	1.34 [0.61, 2.06]	0.15	>0.999
	Training 3	**4.17 [2.36, 5.97]**	**3.48**	**0.020**	**5.36 [4.27, 6.46]**	**4.05**	**<0.001**	1.36 [0.66, 2.06]	0.18	>0.999
	Assessment	1.74 [0.90, 2.59]	1.05	0.907	2.79 [1.91, 3.66]	1.47	0.450	1.75 [0.83, 2.67]	0.57	0.923
	Retention	1.43 [0.65, 2.21]	0.74	0.978	2.31 [1.77, 2.85]	0.99	0.812	1.71 [0.80, 2.61]	0.52	0.944

**TABLE III:** Fixed-Effects Estimates for Mixed-Effects Learning Curve

Parameter	Value—Solo		Difference (LS - Solo)		Difference (MS - Solo)	
	Estimate [95% CI]	p-value	Estimate [95% CI]	p-value	Estimate [95% CI]	p-value

Asymptote (α)	**34.04 [29.90, 38.18]**	**<0.01**	4.08 [−1.76, 9.93]	0.17	−3.09 [−8.88, 2.70]	0.30
Train Offset ($\beta_{t}$)	**2.44 [0.04, 4.85]**	**0.05**	**−5.07 [−8.45, −1.69]**	**<0.01**	2.44 [−0.87, 5.74]	0.15
Total Change (c)	**−10.61 [−14.46, −6.77]**	**<0.01**	−0.33 [−5.81, 5.15]	0.91	−3.02 [−8.44, 2.41]	0.28
Learn Rate (ℓ)	**0.35 [0.20, 0.52]**	**<0.01**	0.15 [−0.14, 0.45]	0.31	0.15 [−0.10, 0.41]	0.24
Forgetting ($c_{r}$)	**−4.26 [−7.54, −0.99]**	**0.01**	**4.65 [1.04, 8.27]**	**0.01**	−0.70 [−5.53, 4.12]	0.78
Relearn Rate ($\ell_{r}$)	0.39 [−0.06, 0.83]	0.09	**−0.64 [−1.11, −0.18]**	**0.01**	2.89 [−13.95, 19.73]	0.74

**Note:** The solo group is treated as the reference group; the table therefore reports the estimated value for the solo group and the relative differences for the less skilled (LS) and more skilled (MS) dyad groups.
